# Complete genome sequence of the type strain of *Mycobacterium montefiorense*, strain ATCC BAA-256

**DOI:** 10.1128/mra.00476-24

**Published:** 2024-07-02

**Authors:** Takeshi Komine, Mitsunori Yoshida, Mitsuhiko P. Sato, Yoshinori Hasegawa, Yoshihiko Hoshino, Shinpei Wada, Hanako Fukano

**Affiliations:** 1 Department of Mycobacteriology, Leprosy Research Center, National Institute of Infectious Diseases, Tokyo, Japan; 2 Kazusa DNA Research Institute, Chiba, Japan; 3 Laboratory of Aquatic Medicine, School of Veterinary Medicine, Nippon Veterinary and Life Science University, Musashino, Tokyo, Japan; University of Southern California, Los Angeles, California, USA

**Keywords:** *Mycobacterium montefiorense*, complete genome, Hi-Fi sequencing, nontuberculous mycobacteria

## Abstract

*Mycobacterium montefiorense*, a nontuberculous mycobacterium, is a causative agent of mycobacteriosis in aquatic animals, its type strain *M. montefiorense* ATCC BAA-256 being isolated from a moray eel. In this study, we report the complete ATCC BAA-256 genome sequence with a 5,693,452-bp-containing circular chromosome, 65.2% GC content, and 5,407 coding sequences.

## ANNOUNCEMENT

*Mycobacterium montefiorense* is a slow-growing nontuberculous mycobacterium and mycobacteriosis causative agent in fish and salamanders (1 – 3). ATCC BAA-256, the *M. montefiorense* type strain, was isolated from a granulomatous skin lesion of a moray eel (1, 2). Only a few draft genome sequences of *Mycobacterium montefiorense* have been reported so far (4, 5). The complete genome presented herein could potentially contribute to future genetic studies of mycobacterial species.

We streaked a −80°C-frozen 20% glycerol stock of single-cloned ATCC BAA-256 onto Middlebrook 7H10 agar supplemented with 10% BBL Middlebrook oleic acid-albumin-dextrose-catalase enrichment (Becton, Dickinson and Company, USA) and incubated it at 25°C for 3 months. We collected ATCC BAA-256 colonies on the agar (approximately 1.0 × 10^10^ CFU) and extracted high-molecular-weight DNA according to a previously described method (6) with slight modifications: We used twice the volume of lysates and 500 µL TE buffer (10 mM Tris-HCl, 1 mM EDTA) and adjusted the final concentration of RNase A to 20 µg/mL. We sheared the genomic DNA to 10–20 kb using a Covaris g-TUBE (Covaris, USA) and prepared sequencing libraries using the SMRTbell Prep Kit (version 3.0; Pacific Biosciences, USA), size selected (>5 kb) using a BluePippin instrument (Sage Science, USA). We sequenced the libraries using the PacBio Revio system (Pacific Biosciences) and obtained 709,710 HiFi reads (>*Q*20) with an average length and median quality value of 7,555 bp and *Q*44, respectively. We converted the HiFi reads using bam2fastq (https://github.com/jts/bam2fastq) and filtered them by read length (≥14,000 bp) with filtlong v0.2.1 (https://github.com/rrwick/Filtlong). We *de novo* assembled the selected 7,196 reads with sum and average lengths of 111,214,722 and 15455.1 bp, respectively, and an *N*_50_ value of 15,056 bp using Flye v2.9.3-b1797 (7). Default parameters were used for all software unless otherwise specified.

We generated a 5,693,452-bp circular chromosomal contig with a GC content and average coverage of 65.2% and 19×, respectively. We performed automated annotation using the FAST Annotation and Submission Tool of the DNA Data Bank of Japan (DFAST; https://dfast.ddbj.nig.ac.jp/), identifying 5,407 coding sequences, 3 rRNA genes, and 54 tRNA genes.

## Data Availability

The raw sequencing data are available in the DDBJ Sequence Read Archive under the accession number DRR541494, BioSample accession number SAMD00467631, and BioProject accession number PRJDB13312. The complete genome sequence was submitted to the DDBJ at AP031386.
